# A new approach to haemoglobin oxygen affinity research at high altitude: Determination of haemoglobin oxygen dissociation curves and 2,3-bisphosphoglycerate in an experimental human crossover hypoxic chamber study

**DOI:** 10.1007/s00421-025-05806-1

**Published:** 2025-05-02

**Authors:** Simon Woyke, Herbert Oberacher, David Plunser, Christoph Siebenmann, Rachel Turner, Ivo B. Regli, Maja Schlittler, Giacomo Strapazzon, Hermann Brugger, Mathias Ströhle, Thomas Haller, Hannes Gatterer

**Affiliations:** 1 Department of Anaesthesiology and Critical Care Medicine, Medical University of Innsbruck, Anichstraße 35, 6020 Innsbruck, Austria; 2 Institute of Mountain Emergency Medicine, Eurac Research, Bolzano, Italy; 3 Institute of Legal Medicine and Core Facility Metabolomics, Medical University of Innsbruck, Innsbruck, Austria; 4 Department of Physiology and Medical Physics, Institute of Physiology, Medical University of Innsbruck, Innsbruck, Austria

**Keywords:** P50, Half saturation pressure, High-altitude physiology, Acclimatisation

## Abstract

**Purpose:**

During rapid exposure to hypobaric hypoxia (HH), arterial oxygen tension and haemoglobin oxygen saturation decrease. The oxygen dissociation curve (ODC) describes the relationship of oxygen tension and haemoglobin oxygen saturation. Previous methods for ODC determination are mostly limited to standard conditions (40 mmHg PCO_2_, 37 °C), and measurements of 2,3-bisphosphoglycerate (2,3-BPG) and adenosine triphosphate (ATP) are omitted. This study aimed to investigate hypoxia-induced changes on the ODC in a strictly controlled hypobaric chamber setting utilising a novel method for ODC determination, incorporating innovative 2,3-BPG and ATP measurements.

**Methods:**

In a randomised crossover design, ten healthy males completed two 4-day sojourns, one in HH (3,500 m) and one in normoxia (NX, 262 m). ODCs were determined from venous blood at 96 h using a highly customisable high-throughput microplate reader method. Haemoglobin half saturation pressure (P50) was measured for a standardised CO_2_ tension of 40 mmHg (P50_st_) and individual end-tidal CO_2_ tensions (P_et_CO_2_) (P50_act_). 2,3-BPG and ATP were determined by liquid chromatography–tandem mass spectrometry.

**Results:**

P50_st_ were increased in HH compared to NX but missed statistical significance (28.3 ± 2.0 mmHg vs. 26.8 ± 1.9 mmHg; *p* = 0.054), whilst P50_act_ was similar in HH and NX (26.4 ± 1.6 mmHg vs. 26.1 ± 2.7 mmHg; *p* = 0.360) despite reduced P_et_CO_2_ (31.0 ± 2.1 mmHg vs. 38.4 ± 2.5 mmHg; *p* < 0.001). 2,3-BPG and pH were significantly increased in HH compared to NX (16.8 ± 1.6 µmol/gHb and 20.5 ± 1.1 µmol/gHb, *p* < 0.001; 7.36 ± 0.01 versus 7.39 ± 0.02, *p* < 0.001).

**Conclusion:**

The 2,3-BPG increase after 96 h in HH compensates the effect of hypoxia-induced decrease in P_et_CO_2_/increase in pH on the ODC.

## Introduction

The oxygen dissociation curve (ODC) describes the reversible, non-linear binding of oxygen to haemoglobin (Mairbaurl and Weber 2012; Severinghaus 1979). In addition to some agents, such as 5-hydroxymethylfurfural (Woyke et al. 2021), voxelotor (Stewart et al. 1985) or carbon monoxide (Gatterer et al. 2024), there are four main physiological effectors of the ODC, namely temperature, pH, partial pressure of carbon dioxide (PCO_2_) and 2,3-bisphosphoglycerate (2,3-BPG). The two main parameters used to describe the ODC are the P50 (oxygen partial pressure at which 50% of haemoglobin is saturated with oxygen) and the Hill coefficient (steepest slope in the Hill plot, a measure of cooperativity of the Hb-subunits) (Imai 1982). CO_2_ acts as an effector to the ODC in two different ways: first by lowering the pH (respiratory acidosis), and second by binding directly to the N-terminal end of the haemoglobin β-chains (1). The combination of both effects is known as the Bohr effect, causing a right shift of the ODC (decrease in oxygen affinity) when CO_2_ or H^+^ are elevated. Synthesis of 2,3-BPG by the Rapoport–Luebering shunt in glycolysis is also pH-sensitive and stimulated by intracellular alkalosis. 2,3-BPG results in a right shift of the ODC, thereby improving oxygen delivery to the tissue (Mairbaurl and Weber 2012). A left shift of the ODC (increased Hb-O_2_ affinity) indicates an improved oxygen uptake in the lungs with a possible deterioration of oxygen unloading at the tissue level, whilst a right shift of the ODC (decreased Hb-O_2_ affinity) indicates an impaired pulmonary oxygen uptake with the possibility of enhanced oxygen unloading at the tissue level. Under certain circumstances, e.g., hypoxia in high altitude, during exercise or in hypoxemia due to respiratory illness, it is currently under debate whether an increased or decreased Hb-O_2_ affinity might be beneficial (Dempsey 2020; Shepherd et al. 2019; Woyke et al. 2020).

There is a long history of research into the effects of different levels of hypobaric hypoxia (HH) on the ODC. As early as 1923, Barcroft investigated the oxygen affinity of Hb in Peruvian natives living at altitudes between 3,500 and 5,500 m and found a left shift of the ODC with lower P50s (oxygen partial pressure at which 50% of haemoglobin is saturated with oxygen) in people living at 4,300 m compared to those living at sea level (Barcroft 1923). This finding was also recently confirmed for Han Chinese and Tibetan people (Li et al. 2018). However, evidence for acute effects of HH exposure on ODC determination remains varied, potentially related to the severity of altitude dose and different definitions of P50.

Early work from Humpeler et al. (1979) investigated the ODC in eight male volunteers after 5 days at moderate altitude (1,700 m) and observed a left shift of the ODC compared to sea level. This left shift was explained by respiratory alkalosis and a delayed increase in 2,3-BPG within several days. Ten days later, 2,3-BPG was profoundly increased and led to a right-shifted ODC despite continued respiratory alkalosis (Humpeler et al. 1979). However, a further study involving 12 subjects residing for 13 days at 2,300 m, observed that P50 (determined by the two-point method) was already elevated on day one, likely due to increases in 2,3-BPG (Mairbäurl et al. 1986). At higher altitude (i.e. 4,500 m), Mairbäurl et al. reported P50 (measured at a standardised arterial PCO_2_ of 40 mmHg (P50_st_)) to be increased in 11 males, whereas P50_act_ (including actual end-tidal PCO_2_ values) remained stable (Mairbäurl et al. 1985a). Whilst in this study, 2,3-BPG was initially increased after 1 day (Mairbäurl et al. 1985a), in a subsequent study from the same group, 2,3-BPG was found to plateau only after 4 days (Mairbäurl et al. 1985b). In contrast, without measuring 2,3-BPG levels and maintaining standard PCO_2_ levels, Balaban et al., reported a left shift of the ODC when exposing participants to 4,000 m (Balaban et al. 2013). At very high altitude (> 5,000 m), the effect of a reduced PCO_2_ due to hyperventilation is known to outweigh the effect of 2,3-BPG (Winslow et al. 1984; Wagner et al. 2007). This has been shown by a comparison of in vivo P50 (PCO_2_ and 2,3-BPG effect) and standard P50 (only 2,3-BPG effect) (Wagner et al. 2007). The majority of these studies are performed in the field in natural altitude, where barometric pressure is varying and other conditions, such as climatic or wind, might contribute to outcomes (Millet and Debevec 2020). Studying the sole effect of hypobaric hypoxia on ODC and 2,3-BPG necessitates strict control of confounding conditions.

For the study of Hb-O_2_ affinity, especially when related to high altitude, the determination of 2,3-BPG and ATP is essential (Mairbaurl and Weber 2012). Previous 2,3-BPG measurements have often been performed using enzymatic assays. However, production of these assays has been discontinued, leading to recent publications detailing Hb-O_2_ affinity without 2,3-BPG measurements (Böning et al. 2021). Equally, ATP (another prime effector of the ODC), is measured less frequently still. Thus, alternative approaches for measuring 2,3-BPG and ATP levels are needed. We and others have demonstrated that liquid chromatography–tandem mass spectrometry (LC–MS/MS) appears to be a suitable tool to fill this gap (Kim et al. 2017; Woyke et al. 2022). Equally, the majority of previous studies, particularly including the in vivo determination of P50, are based on a one- or two-point method to determine the sigmoidal shape of the ODC. Therefore, a recently presented method for high-throughput ODC measurements utilising a conventional microplate reader with a customisable experimental setup surely opens new avenues for high-altitude-related Hb-O_2_ affinity research.

In this study, we aim to investigate the HH (3,500 m)-induced changes in P50 as well as the blood concentrations of 2,3-BPG and ATP in a highly controlled experimental hypobaric hypoxic chamber setting applying innovative analytical methods.

## Methods

This study was approved by the Ethics Committee of the Hospital of Bolzano (vote nr.: 70–2019).

In a randomised crossover design, 11 male volunteers (25 ± 4 years, 72 ± 12 kg, 181 ± 8 cm) completed two 4-day sojourns in a hypobaric chamber (terraXcube, EURAC Research, Bolzano, Italy), one in normoxia (NX, 741 mmHg) and one in HH (493 mmHg). The subjects were in good health, non-smokers, not accustomed to heavy daily exercise and without recent (2 weeks) sojourn to altitude > 2,000 m. During the two chamber sojourns, subjects reproduced their normal daily step counts and adhered to a standardised dietary and fluid intake, as previously described by Schlittler et al. (Schlittler et al. 2021). After the fourth night in NX and HH (96 h), blood samples were collected from a peripheral arm vein into heparinised syringes and immediately stored on ice to reduce metabolic changes. At the time of blood sampling, the subjects were fasted and had remained supine for at least 30 min prior. End-tidal partial pressure of carbon dioxide (P_et_CO_2_) was obtained from a pulmonary gas exchange monitor (MediPines Gas Exchange Monitor, MediPines Corp., Yorba Linda, USA). A 30 µl aliquot was taken and frozen at −80 °C for later determinations of 2,3-BPG and ATP. Lactate concentration was determined separately using a blood gas analyser (Radiometer ABL90 flex).

ODC measurements were carried out within 4 h of blood sampling during which time whole blood parameters were found to be stable (Woyke et al. 2021). Both standard ODC (P50_st_) with a PCO_2_ of 40 mmHg at 37 °C and an actual ODC (P50_act_) with a PCO_2_ adjusted for each subject according to their individual P_et_CO_2_ at 37 °C was recorded. The Hill coefficient, a measure of cooperativity of the Hb-subunits, was determined as the steepest slope in the Hill plot. Gas mixtures were produced volumetrically. A minimum of 20 ODCs were measured and P50 results averaged for each subject, and an internal standard solution for calibration was used as previously described (Woyke et al. 2021). P50_st_ was measured in a single gas-flow microplate with an internal standard solution, P50_act_ was recorded using the multiple gas-flow microplate with individualised PCO_2_ for each subject (Woyke et al. 2021).

Concentrations of 2,3-BPG and ATP were determined employing a recently published LC–MS/MS workflow (Woyke et al. 2022). The method enabled reliable quantitative analyses of the two targets in the range 50–10,000 μg/mL. Isotopically labelled analogues (ATP-^13^C_10_ and 2,3-BPG-^13^C_3_) were used as internal standards. Sample processing of blood samples (10 µl) involved protein precipitation with methanol and dilution. The LC–MS/MS system consisted of a 1100 series HPLC pump (Agilent, Waldbronn, Germany), a CTC-PAL autosampler (CTC Analytics AG, Zwingen, Switzerland), and a QTrap 4000 mass spectrometer (Sciex, Framingham, MA, USA). Separation was performed on an InfinityLab Poroshell 120 HILIC-Z column (2.7 µm, 2.1 × 100 mm, PEEK lined; Agilent, Santa Clara, USA) by applying a linear gradient of 82–5% acetonitrile in 10 mM ammonium acetate (pH 9.0) containing 2.5 µM medronic acid within 7 min. The flow rate was set to 250 µl/min. The column temperature was 30 °C. The injection volume was 10 µL. Mass spectrometric detection was performed with electrospray ionisation in negative ion mode and multiple reaction monitoring.

Student’s *t* test (one-tailed) was used to compare means. Excel (Microsoft Office 2016, Microsoft Corp.) was used for data collection, calculation, and analysis. *p* < 0.05 was considered statistically significant. Results are expressed as arithmetic mean ± standard deviation (SD).

## Results

Subject’s characteristics are summarised in Schlittler et al. (Schlittler et al. 2021). Due to a delayed storage of one blood sample at room temperature in HH, one participant had to be excluded from analysis.

Haemoglobin concentration (Hb) and haematocrit were significantly increased in HH compared to NX in this study, as previously reported (Schlittler et al. 2021). Significant changes in acid base status from venous blood samples are shown in Table 1.

**Table 1:** Results of blood gas analysis at 96 h in normoxia and hypobaric hypoxia

	NX	HH	
pH	7.36 ± 0.01	7.39 ± 0.02	p < 0.001
P_v_CO_2_ [mmHg]	50.3 ± 3.81	40.3 ± 3.05	p < 0.001
Lactate [mmol/l]	0.68 ± 0.19	1.32 ± 0.51	p = 0.001

*NX* normoxia (741 mmHg), *HH* hypobaric hypoxia (493 mmHg), *P*_*v*_*CO*_*2*_ venous carbon dioxide partial pressure. Data are presented as mean ± SD; *n* = 10; *p* values result from time-point comparisons between HH and NX by analyses by Student’s t test (one-tailed). *p* values < 0.05 are considered significant

**Fig. 1:**
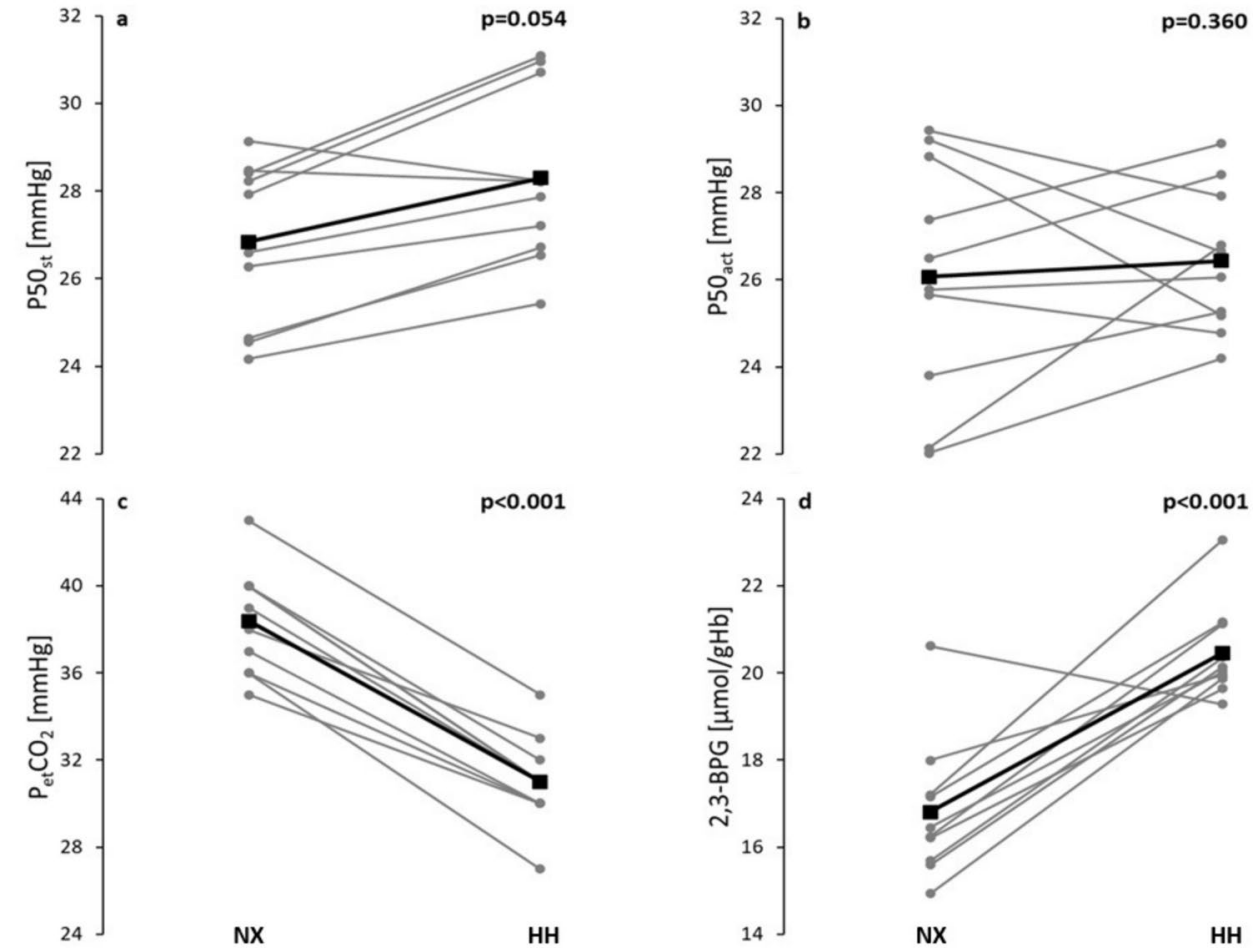
Comparison of **a** standard P50 with a PCO_2_ of 40 mmHg at 37 °C (P50_st_), **b** actual P50 with a PCO_2_ adjusted for each subject according to their individual P_et_CO_2_ at 37 °C (P50_act_), **c** end-tidal carbon dioxide partial pressures (P_et_CO_2_), and **d** 2,3-bisphosphoglycerate levels in whole blood (2,3-BPG), between normoxic (NX, 741 mmHg) and hypobaric hypoxic (HH, 493 mmHg) conditions after 96 h of exposure. Black symbols represent group means and grey symbols individual values (*n* = 10). *p* values result from time-point comparisons between HH and NX by Student’s *t* test (one-tailed) analyses. *p* values < 0.05 are considered significant

P_et_CO_2_ levels decreased significantly from 38.4 ± 2.5 mmHg in NX to 31.0 ± 2.1 mmHg in HH (*p* < 0.001), similarly to the P_v_CO_2_ levels measured in BGA (Table 1). The changes in P50_st_ in HH compared to NX were not statistically significant (26.8 ± 1.9 mmHg NX and 28.3 ± 2.0 mmHg HH; *p* = 0.054) (Fig. 1). Equally, P50_act_ remained stable between the two conditions (26.1 ± 2.7 mmHg NX and 26.4 ± 1.6 mmHg HH; *p* = 0.360). 2,3-BPG was significantly increased in HH compared to NX (16.8 ± 1.6 µmol/gHb NX and 20.5 ± 1.1 µmol/gHb HH; *p* < 0.001).

Figure 1 shows the results for each individual. ATP was decreased in HH (4.1 ± 0.5 µmol/gHb NX and 3.6 ± 0.5 µmol/gHb HH; *p* = 0.042). Hill coefficients did not change, despite of changes in P50 (2.51 ± 0.08 NX and 2.55 ± 0.09 HH; *p* = 0.130).

## Discussion

The aim of this study was to investigate the short-term HH induced changes in P50 as well as the blood concentrations of 2,3-BPG and ATP using innovative analytical methods. Rather than comparing in vivo with in vitro measurements, as frequently done, this study replicated the individual in vivo situation in an in vitro method for ODC determination. The possibility of adjusting PCO_2_ to individual levels and the high-throughput character of the ODC method is fit to adequately investigate combined effects of PCO_2_ and 2,3-BPG/ATP on the ODC. In addition, a convenient workflow for the quantitation of 2,3-BPG and ATP that enables direct and valid determination of concentrations in whole blood samples stored at −80 °C might positively impact the inclusion of these parameters into further high-altitude research.

The main findings of this study are that after 4 days (96 h) in HH (3,500 m), a rise in 2,3-BPG concentration decreases Hb-O_2_ affinity, indicated by an increase in P50_st_. 2,3-BPG levels, in turn, increased due to a small alkalinisation, as shown by BGA analysis. However, P50_act_ remains stable indicating that elevated 2,3-BPG levels compensate for the hypoxia-induced respiratory alkalosis.

The study of Hb-O_2_ affinity at high altitude requires a specific combination of approaches. For instance, to evaluate the effect of HH alone, studies need to be conducted under well-controlled conditions to minimise the impact of potential confounding factors (e.g. nutrition, exercise, and environmental effects such as cold or heat). In addition, transportation and storage of blood samples needs to be considered to ensure accurate and precise results. Appropriate research equipment must be available. In the context of field research at high altitude, this ideal is difficult to obtain. However, these criteria can be met in chamber studies.

In the present chamber study, diet, fluid balance and exercise were matched between NX and HH conditions, which should have minimised potential confounding factors. Moreover, based on the design of the in vitro ODC method used, several methodological modifications have been possible, in particular the adjustment to individual P_et_CO_2_ levels. Equally, this allows new approaches to P50 measurements, where P50 is not only measured under standardised conditions (37 °C, 40 mmHg PCO_2_), but also with regard to the in vivo condition with reduced PCO_2_. This is critical as changes in PCO_2_ due to hyperventilation can strongly influence P50. By enabling measurements under in vivo conditions, the present data principally confirm the results of Mairbäurl et al. (Mairbäurl et al. 1985a) and Wagner et al. (Wagner et al. 2007), but also show considerable inter-individual differences. At altitudes up to approximately 5,000 m, the effect of 2,3-BPG seems to mitigate the effect of PCO_2_, resulting in an unchanged P50. Conversely, at altitudes > 5,000 m, the effect of 2,3-BPG may be outweighed by extreme hypocapnia following hyperventilation, inducing a left shift (Winslow et al. 1984). However, this study investigated only a time period of 4 days, whilst changes requiring a longer period might be overlooked. Further studies investigating long term effects are needed.

Whilst this study provides valuable insights, it is important to acknowledge its limitations, which may affect the generalisability and interpretation of the findings. This study was part of a larger investigation on the adaptation of humans to moderate altitude (Schlittler et al. 2021; Gatterer et al. 2021; Tremblay et al. 2020; Regli et al. 2021). Thus, potentially conflicting interventions, particularly a CO-rebreathing protocol for all subjects, has to be taken into account as COHb affects Hb-O_2_ affinity. This intervention was scheduled the evening before blood collection for ODC determination (Schlittler et al. 2021). As a consequence, COHb was still increased the next day (NX 2.49 ± 0.40% and HH 2.45 ± 0.31%; Pp = 0.806). A significant effect on the present results, however, is unlikely as the increase was very modest and similar in NX and HH. Furthermore, PCO_2_ was obtained from P_et_CO_2_ measurements and not directly from arterial blood gases, probably limiting accuracy.

In conclusion, in this study, we demonstrate the adaptability and applicability of a new and advanced method for high-throughput ODC determination for high-altitude-related Hb-O_2_ affinity research. Complementary information on 2,3-BPG and ATP concentrations was obtained by analysing blood samples stored at -80 °C with a convenient and validated LC–MS/MS workflow. With this innovative experimental setup, we were able to show that the 2,3-BPG increase after 96 h at 3,500 m counteracts the effect of the hypoxia-induced respiratory alkalosis on the ODC. 

## Data Availability

The data of this study are available from the corresponding author upon reasonable request.

## Abbreviations

2,3-BPG: 2,3-Bisphosphoglycerate
ATP: Adenosine triphosphate
Hb: Haemoglobin
HH: Hypobaric hypoxia
LC–MS/MS: Liquid chromatography–tandem mass spectrometry
NX: Normoxia
ODC: Oxygen dissociation curve
P50: Half saturation pressure of haemoglobin
PCO2: Partial pressure of carbon dioxide
